# Encapsulation of *B. bassiana* in Biopolymers: Improving Microbiology of Insect Pest Control

**DOI:** 10.3389/fmicb.2021.704812

**Published:** 2021-08-16

**Authors:** Ana Paula Felizatti, Roberta Maria Manzano, Inajá Marchizeli Wenzel Rodrigues, Maria Fátima das Graças Fernandes da Silva, João Batista Fernandes, Moacir Rossi Forim

**Affiliations:** Laboratory of Natural Products, Universidade Federal de São Carlos, São Carlos, Brazil

**Keywords:** *Beauveria bassiana*, Spray-drying, microencapsulation, *Spodoptera cosmioides*, biopolymers

## Abstract

The fungus *Beauveria bassiana* is widely used for pest control; however, biostability and dispersion for broth pulverization are limiting factors for its application in the field. In this context, formulation techniques such as microencapsulation are viable alternatives. The aim of this work is to optimize *B. bassiana* formulations by spray dryer and evaluate its stability and biological activity against *Spodoptera cosmioides* compared to ionic gelatinization formulations. The fungus was biocompatible with all evaluated biopolymers (lignin, cellulose, starch, humic substances, and alginate). The encapsulation by spray drying was optimized by factorial design in an inlet and outlet air temperature of 120°C and 68°C, respectively; aspirator rate of 35 m^3^·h^−1^, feed flow rate of 12 mL·min^−1^; and drying gas flow at 35 L·h^−1^. The ionic gelation capsules were obtained using a 0.5% quantity of conidia in a 1% sodium alginate solution dropped into a 0.5 mol·L^−1^ CaCl_2_ solution using a peristaltic pump. Spray drying provided smaller microcapsules than those by ionic gelation. Both techniques produced more stable conidia when exposed to temperature and UV-radiation than non-formulated *B. bassiana*. The formulations prepared by spray drying showed gains at aqueous dispersion. Biological assays against *Spodoptera cosmioides* showed a mortality rate of up to 90%. These results demonstrate the suitability of encapsulating *B. bassiana* conidia stably in aqueous dispersion without loss of viability and virulence.

## Introduction

The control of agricultural pests has been conducted mainly by synthetic chemical insecticides. This control system has been efficient in promoting productivity gains every year. However, it is responsible for several harmful damages to the environment (Sharma et al., 2019). Prolonged exposure to conventional synthetic pesticides raises occupational risks, disturbances to human health, and harmful impacts on ecosystems and the environment. More specifically, these substances may also attack non-target species and/or lead to the emergence of new pests (Keshani et al., 2015). Lastly, several studies highlight the negative effects of synthetic pesticide use on human health (Heckel, 2012; Asghar and Malik, 2016; Bourguet and Guillemaud, 2016; Op de Beeck et al., 2017; Sharma et al., 2019).

In the search for sustainable agriculture, there is a plethora of alternative products that have been continuously evaluated to replace conventional agrochemicals, such as agricultural biodefensives for biological control (Mossa, 2016). The entomopathogenic fungus *Beauveria bassiana* (Bals.) Vuill. has a prominent position among these so-called green insecticides. It is a broad-spectrum biological control agent against insects considered agricultural pests. Alali et al. (2019) and Mascarin and Jaronski (2016) demonstrated the virulence from several *B. bassiana* strains on arthropods, with a mortality rate of up to 90%. It has shown promising results in controlling pests such as *Ephestia kuehniella, Bemisia tabaci, Metamasius hemipterus, Hyphotenemus hampei, Anastrepha fraterculus, Tetranychus urticae, Nezara viridula, Diaphorina citri*, and *Thaumastocoris peregrinus*, which are commonly found in coffee, eucalyptus, soy, citrus, etc.

Several entomopathogenic microorganisms are already produced on a commercial scale; however, their agricultural use still faces limitations due to lack of stability to temperature and UV-light. These abiotic factors not only compromise their biological efficiency but also present problems with storage, transport, and field application (Parra, 2014; Sinha et al., 2016). The optimum temperature for the development of these microorganisms in the field in order to keep its virulence is ~25°C (Bugeme et al., 2008). Under Brazilian climatic conditions, this temperature is easily exceeded in agricultural fields and storage places, compromising their viability and efficiency. García-Estrada et al. (2016) highlighted that the low shelf life and field stability of microbial formulations compromise their commerce and acceptance.

The demand for sustainable techniques for integrated pest management requires methods to stabilize biopesticides, emphasizing the conservation of microbial propagation features and the upkeep of their genotypic and phenotypic characteristics. Microbial microencapsulation may provide the conservation of these characteristics. Muñoz-Celaya et al. (2012) demonstrated that the process of encapsulation of *Trichoderma harzianum* in polymeric carbohydrates showed a 330-fold increase in shelf life. Maruyama et al. (2020) also demonstrated that *T. harzianum* encapsulation increased the potential for biological control of *S. sclerotiorum*. Lastly, Qiu et al. (2019) developed methods for encapsulating *Metarhizium anisopliae* in gelatin, showing gains in UV-light protection and shelf life, keeping bioactive microorganisms against *Solenopsis invicta*.

The encapsulation processes include the trapping of an active compound in a matrix (in this case, microbial conidia), ideally with gains in biotic and abiotic protection within the microenvironment generated by the encapsulating material (Vemmer and Patel, 2013). Another advantage is the possibility of controlling the release of encapsulated conidia by environmental activators. Maruyama et al. (2020) demonstrated that encapsulation influenced the release kinetics of *T. harzianum*, increasing its bioavailability in comparison to non-encapsulated fungus.

The use of biodegradable materials in formulations is ecologically advantageous and biologically safe. Biodegradable materials often do not present harmful residues to the environment, nor toxicity, facilitating registration and approval for use (Sawalha et al., 2011; Rajeswari, 2017). Examples of available biodegradable compounds are starch, chitosan, alginate, gum arabic, cellulose, lignin, gelatin, etc. Each material has different properties regarding stability, solubility, swelling/erosion, release kinetics, biocompatibility, etc. These characteristics may affect microbial development. Therefore, the association/choice of microorganism and its encapsulation material should be investigated, evaluating its benefits and compatibility with the encapsulation process (Wandrey et al., 2010; Silva et al., 2014).

Among the encapsulation techniques, dripping methods have a stand out position. They form droplets through a nozzle, either by spray drying or ionic gelation (Vemmer and Patel, 2013). The spray drying process works by nebulizing the dispersion medium that contains the polymer and active compounds into a hot air stream, evaporating the solvent during its way to the storage vial, obtaining dried particles (Santos et al., 2018). Ionic gelation promotes the formation of a highly ordered hydrogel encapsulation structure when a solution containing the biopolymer and the active agent is dropped into a crosslinking solution containing divalent cations (Ching et al., 2017). Both techniques can be used for thermally sensitive compounds, such as essential oils, pharmaceuticals, and biological supplies (Vemmer and Patel, 2013; Rigon and Zapata Noreña, 2016; Arpagaus et al., 2018; Veiga et al., 2019). Liu and Liu (2009) demonstrated that the spray drying encapsulation of *B. bassiana* conidia maintained 80% of viability for up to 6 months when stored at low temperature (4°C). However, Rosas-García et al. (2001) demonstrated losses in *B. bassiana* viability regardless of the type of evaluated polymer in spray dryer processes. As a result, there is a lack of studies that help understand the encapsulation process for entomopathogenic conidia.

Therefore, the objective of this work was to establish encapsulation parameters for the entomopathogenic microorganism *B. bassiana* via spray drying using biodegradable materials. We also investigated the maintenance of microbial viability and virulence using *Spodoptera cosmioides* caterpillars as a model. The spray dryer encapsulation was evaluated comparatively to the ionic gelation process.

## Materials and Methods

### *B. bassiana* Conidia

Pure *B. bassiana* conidia was provided by Biocontrol—Controle Biológico (Sertãozinho, SP-Brazil), and were kept at −12°C until use. The *B. bassiana* conidia (strain IBCB 66) were isolated initially from *Hypothenemus hampei* (coffee borer beetle) and preserved in mineral oil at the “Oldemar Cardim Abreu” Entomopathogenic Microorganisms Collection in the Campinas Experimental Center at the Biological Institute, São Paulo, Brazil. This strain is a commercial product widely applied in Brazilian crops (Ribeiro et al., 2012). The commercial powder of *B. bassiana* was initially evaluated in terms of germination before any experiment was carried out. For this we incubated the microbiological powder for 7 days at 25.5°C ± 0.5°C in B.O.D (Biochemical Oxygen Demand, Eletrolab, EL101, Brazil), under a 14-h light photoperiod, using PDA (Potato-Dextrose-Agar, KASVI, Brazil) in 9-cm-diameter Petri dishes. In order to assure a standard stock, the quantification of spores per milligram of commercial powder was carried out with the aid of a hemocytometer (Neubauer chamber, KASVI, Brazil) in sextuplicates, as described in section Quantification of *B. bassiana* Conidia.

### Materials to Encapsulation

We evaluated six substances as encapsulation agents, soy oil, corn starch, cellulose, lignin, alginate, and humic acid. The soy oil and starch were purchased in a local supermarket in the city of São Carlos, state of São Paulo, Brazil. Cellulose and alginate were bought from Vetec Química (Rio de Janeiro, RJ–Brazil). The humic acid and lignin were provided by Agrolatina Biotecnologia AS (Rincão, SP–Brazil), and Suzano Papel e Celulose (Suzano, SP–Brazil), respectively. The CaCl_2_ used on ionic gelation was purchased from Sigma-Aldrich, and all the growth media are from Kasvi do Brasil (São José dos Pinhais, PR–Brazil).

### Quantification of *B. bassiana* Conidia

Initially, we quantified the total *B. bassiana* conidia in the commercial product before using it in the formulations and stability studies. The quantification was performed with a hemocytometer (Neubauer chamber, Kasvi, Brazil) under an optical microscope on a microscope slide using sticky tape. In this case, a sticky tape was placed under the dish containing *B. bassiana* and put on a microscopy slide containing Amman's lactophenol dye (Newprov, Brazil) (Rice and Cogburn, 1999). Samples were evaluated with a LX500Phase Contrast optical microscope (Labomed, Los Angeles, CA, USA) equipped with a IVU5000 digital video camera and magnified up to 400× for counting conidia (Rice and Cogburn, 1999). Microscopy images can be seen in Supplementary Figure 1.

### Biocompatibility Assay Between *B. bassiana* and Biopolymers

Previously, we evaluated the biocompatibility of alginate and cellulose to *B. bassiana* (Wenzel Rodrigues et al., 2017). In short, conidia were inoculated in a culture medium containing the compound of interest, and the evaluation of its growth was performed using the protocol described by Rice and Cogburn (1999). In this work, we expanded the biocompatibility study for soy oil, humic acids, corn starch, and pine lignin. These results contributed to the initial selection of working biopolymers.

This part of the study was carried out by inoculating a 10-μl drop of the unformulated microorganism suspension at 10^9^ CFU (colony forming unit)·ml^−1^. These were placed at the center of 9-cm-diameter Petri dishes containing PDA previously sterilized in an autoclave at a pressure of 1 atm at 121°C, for 20 min. The encapsulating agent was incorporated into the growth medium at a temperature of 45 ± 2°C before microbial inoculation at 1.0% and 2.0% (w/v) concentration. We also included a pentabiotic (Zoetis, Campinas, SP–Brazil) at 0.5 g·L^−1^. Petri dishes were kept in a germination chamber (B.O.D.) at temperature of 25.0 ± 0.5°C, in a 12 h photophase for 3 days. We observed the microbial development daily, evaluating the fungal radial growth. At the end of the incubation period, we quantified the conidia and performed a germination analysis using an optical microscope.

We used the Biological Index according to Gonçalves Diniz et al. (2020) to calculate the compatibility factor between conidia and encapsulating agents, using the following equation:

$$\begin{array}{l} BI=\frac{\left[\left(47\times VG\right)+\left(43\times CS\right)+\left(10\times GER\right)\right]}{100} \end{array}$$

Where: *BI*: Biological Index, *VG*: percentage of vegetative growth of the colony compared to control after 3 days, *CS*: percentage of colony sporulation compared to control after 3 days, and *GER*: percentage of conidia germination after 24 h. The *BI* values for product classification are arranged as follows: (a) *BI* ≤ 41: Toxic (*T*), (b) *BI* ≥ 42 and ≤ 66: Moderately toxic (*MD*), and (c) *BI* > 66: Compatible (*C*).

### Preparation of Pre-encapsulation Formulations

After biocompatibility assays, we selected cellulose, lignin, starch, soy oil, and humic acids to develop in the formulations. The search for an ideal formulation was carried out by a fractional factorial design 2^6−2^, totaling 16 formulations, as described in Table 1.

**Table 1:** Variables of fractional factorial design (2^6−2^) for used encapsulating agent mixtures in the formulation processes.

	**Variables**			**Levels**		
				**−1**		**+1**
A	Lignin			Absent		2.5 g·L^−1^
B	Cellulose			Absent		2.5 g·L^−1^
C	Soy oil			Absent		2.5 g·L^−1^
D	Humic acid			Absent		2.5 g·L^−1^
E	Starch			Absent		2.5 g·L^−1^
F	Sodium alginate			Absent		0.1 g·L^−1^
**Experiment**	**Coded levels**					
	**A**	**B**	**C**	**D**	**E**	**F**
1	−1	−1	−1	−1	−1	−1
2	+1	−1	−1	−1	+1	−1
3	−1	+1	−1	−1	+1	+1
4	+1	+1	−1	−1	−1	+1
5	−1	−1	+1	−1	+1	+1
6	+1	−1	+1	−1	−1	+1
7	−1	+1	+1	−1	−1	−1
8	+1	+1	+1	−1	+1	−1
9	−1	−1	−1	+1	−1	+1
10	+1	−1	−1	+1	+1	+1
11	−1	+1	−1	+1	+1	−1
12	+1	+1	−1	+1	−1	−1
13	−1	−1	+1	+1	+1	−1
14	+1	−1	+1	+1	−1	−1
15	−1	+1	+1	+1	−1	+1
16	+1	+1	+1	+1	+1	+1

Compounds were initially solubilized in water under magnetic stirring and later incorporated into the microbial dispersion. The fungal dispersion was prepared in a saline solution containing NaCl 0.15 mol·L^−1^, and Tween 80 (0.001%, w/v), keeping a ratio of 1:7 between conidia and encapsulating agents. This dispersion solution protocol was developed according to Liu and Liu (2009) and Aziz Qureshi et al. (2015). These mixtures were then used in the spray dryer and ionic gelation microencapsulation processes.

### *B. bassiana* Encapsulation Processes

#### Spray Drying Process Optimization

For this process, we used a Büchi B-290 (Büchi Labortechnik AG, Flawil, Switzerland) spray dryer, equipped with a drying chamber of 500 × 100 mm and an atomizing spray nozzle of 0.7 mm. The drying parameters were optimized by a full factorial design 2^4^ according to Antony (2014) with three different independent variables, in two levels: low and high, transformed in (−1) and (+1), respectively. These levels were previously defined in preliminary tests. In total, we randomly performed 16 experiments avoiding systematic error trends (Table 2).

**Table 2:** Optimization by full factorial design 2^4^ for *B. bassiana* formulation using spray drying.

	**Variables**		**Levels**	
			**–1**	**+1**
A	Inlet temperature (°C)		70	120
B	Feed flow rate (L·h^−1^)		0.005	0.01
C	Aspirator rate (L·h^−1^)		301	414
D	Air injection flow (m^3^ h^−1^)		20	35
**Experiment**	**Coded levels**			
	**A**	**B**	**C**	**D**
1	−1	−1	−1	−1
2	+1	−1	−1	−1
3	−1	+1	−1	−1
4	+1	+1	−1	−1
5	−1	−1	+1	−1
6	+1	−1	+1	−1
7	−1	+1	+1	−1
8	+1	+1	+1	−1
9	−1	−1	−1	+1
10	+1	−1	−1	+1
11	−1	+1	−1	+1
12	+1	+1	−1	+1
13	−1	−1	+1	+1
14	+1	−1	+1	+1
15	−1	+1	+1	+1
16	+1	+1	+1	+1

The random error was evaluated by duplicates calculated as described by Pereira and Pereira-Filho (2018). As responses (dependent variables), we calculated the dry powder mass recovery (% w/w) and conidia viability, calculated as follows:

$$\begin{array}{l} Recovery\left(\%,\frac{w}{w}\right)=\frac{Md}{Mn}\times 100 \end{array}$$

Where *M*_*d*_ = mass of material after drying, and *M*_*n*_ = nominal mass value of the material added to the formulation. The calculation of the effects, the probability graphics, and responses were performed using the Octave®4.2.1 software, using the script described by Pereira and Pereira-Filho (2018).

#### Encapsulation by Ionic Gelation

The ionic gelation process was prepared according to Wenzel Rodrigues et al. (2017). The composition of the formulations is also described, as highlighted in Table 1, allowing a comparison between ionic gelation and spray drying. In summary, the previously dispersion of fungi and polymers were added to a concentration of 0.5% (w/v) in a 1% (w/v) sodium alginate solution. After homogenization, the dispersion was dripped into a 0.5 mol·L^−1^ CaCl_2_ solution using a peristaltic pump (TPM 600 55RPM, Watson-Marlow Inc., Wilmington, MA, USA) at 35 drops·min^−1^ flow. The resulting capsules were carefully washed in distilled water, removing CaCl_2_ and alginate excess. The washed spheres were placed on glass dishes at 25°C for 24 h for dehydration.

### Microencapsulated Conidia Thermal Stability, UV-Light Protection, and Dispersion Assays in Aqueous Media

The formulations that showed the best recovery and viability results were selected for dispersion capacity in an aqueous medium, UV-light protection, and thermal stability analysis. This assay was carried out using ultrapure water (Direct-Q 8 UV Milli-Q, Millipore, Molsheim, France) without additives, as we were looking for low-cost and straightforward spraying field methods. The powder formulations were weighed and dispersed in water at a concentration of 1.0 mg·ml^−1^. As a control, we used the commercial non-formulated powder product of *B. bassiana* with the same concentration. The stability of the aqueous dispersion was monitored visually for 2 h observing (or not) the formation of precipitates.

We tested photostability by evaluating the microbial viability of the formulated powdered materials after exposure to UV-light. The compounds were aliquoted in Eppendorf tubes and placed in a glass base inside a wooden chamber, complementarily lined with mirrors (l = 60.0 cm, h = 40.0 cm, and w = 60.0 cm). The wooden chamber was built containing four 15 W G15T8E USHIO lamps (l = 45.0 cm, w = 2.6 cm; USHIO, Tokyo, Japan), with an intense emission spectrum around 306 nm (UV-B region). The energy of the lamps was similar to an irradiance of 6,153 m W·m^−2^. The total radiance inside the wooden chamber was 22.15 kJ·m^−2^·h^−1^. The wooden chamber was equipped with one thermostat and two mini-fan coolers keeping the internal temperature at 25 ± 2°C. As positive and negative controls, samples of non-encapsulated conidia were exposed to UV-light, while others were protected from it, respectively. The UV-light protection was evaluated in exposure periods of up to 48 h.

The thermostability assay was performed with formulation treatment samples arranged in the support tray for Eppendorf tubes in a dry heating bath (KASVI, K80-0, Brazil) protected from light. This test was performed at 60°C for 4 h. As control, we used samples kept under light protection at 4°C.

Aliquots were inoculated in a PDA medium afterwards to check for conidia viability. The dishes were kept for 72 h in a B.O.D. chamber at 25°C. The formulations that showed better stability in the powder were solubilized in an aqueous medium and again subjected to the stability analyses. After the experimental period, the suspensions were inoculated as described above, evaluating conidia viability maintenance.

### Virulence Assay Against *Spodoptera cosmioides*

#### Rearing of *S. cosmioides*

*S. cosmioides* eggs were purchased at PROMIP– Manejo Integrado de Pragas (Limeira—SP, Brazil), and kept in B.O.D. (Biochemical Oxygen Demand, TECNAL, TE-3911, Piracicaba, SP-Brazil) under a photoperiod of 14 h of light and 10 h in the dark at 25°C, until hatching. After the start of the newborn caterpillars' feeding period, their containers were cleaned daily to remove feces. Additionally, insect development was also monitored daily. For feed, we used Greene's artificial diet (Greene et al., 1976). For each 1 L of diet, we initially dissolved agar into 500 ml of water previously sterilized in an autoclave for 15 min. In another container, we solubilized ascorbic acid, formaldehyde, and methylparaben using a homogenizer (RI1364, 400W, Philips Walita, Varginha, MG, Brazil) in a volume of 500 ml of water. After cooling to ~45°C, we mixed both solutions with tetracycline. The final solution was poured into a sterile container for gelation and kept at 4°C until use. Their feed was cut in 2 × 2 cm blocks, ~10 g each, and were supplied as needed.

The L2 stage of caterpillar development (second instar) is the phase with great significant damage to crops due to greater intensity of feeding (Silva et al., 2017). We transferred the caterpillars to larger containers, changing Greene's diet to a new composition, which was prepared using soy protein 50.0 g, wheat germ 50.0 g, beer yeast 31.2 g, casein 25.0 g, beans 62.5 g, ascorbic acid 3.0 g, vitamin complex 5.0 ml, and agar 18.7 g (*q.s*. 1 L water). The rearing of *S. cosmioides* was adapted from Elvira et al. (2010). We did not use microbiological growth-inhibiting agents (tetracycline, formaldehyde, and methylparaben) to avoid compromising fungal development in this modified diet.

#### Inoculation of Formulated *B. bassiana*

*S. cosmioides* caterpillars between stages L3 and L4 were exposed to previously selected *B. bassiana* formulations. These stages are responsible to most crop damage, and hence, the reason why they were selected (Barros et al., 2010; Ayala et al., 2013). The prepared formulations via spray dryer were dispersed in an aqueous medium in an amount of 100 mg·ml^−1^, with 10 μl being dropped onto each caterpillar (1.0 mg of formulated *B. bassiana* in powder). Granular formulations prepared by ionic gelation were weighed and inoculated (1.0 mg) over each diet block.

It is important to highlight that we used the same initial spore concentration for each formulation and technique. Both encapsulation processes changed the final concentration of viable conidia, however, as shown in the results. Therefore, we assayed the same formulated product mass in this step to evaluate the effectiveness of each processed formulation. Since we used the same quantity of viable conidia in each formulation, applying the same mass from formulated products in the assays could show losses during the processes. As such, we were able to compare the formulated and non-formulated conidia in colloidal dispersion by the same number of viable conidia and the same quantities in mass to the different formulations.

For each formulation, two containers with 10 caterpillars were used, totaling 20 caterpillars. We used three controls: (1) positive control (C+), where 1.0 mg of the unformulated *B. bassiana* commercial product was added for analyzing the results of the biological agent, (2) negative control for alginate (CA–) using 1.0 mg of the polymer, free of *B. bassiana*, and (3) negative control (C–) using only the diet in the presence of caterpillars.

We point out that other species*, S. frugiderda* and *S. eridania* were tested in this study. After inoculation, however, a cannibalistic behavior was observed, hindering the maintenance of an adequate number for continuation of the experiments. For this reason, only the results with *S. cosmioides* are reported.

We observed possible daily changes in the morphology of caterpillars after inoculation when removing dead insects. Visual analysis was maintained until the pupal phase of all surviving caterpillars. We determined the mortality of caterpillars, the percentage of formed and defective pupa, average pupal weight, and total mortality, counting non-viable caterpillars and pupae. The insects resulting from viable pupae were segregated into three groups: (1) a group previously treated with formulated conidia by spray drying, (2) a group previously treated with formulated conidia by ionic gelation process, and (3) a control group formed by insects not treated with *B. bassiana*. The pupae were added to glass dishes containing moistened filter paper in order to evaluate the hatching of adults. After the moths hatched, we fed them with a 5% (w/v) honey diet soaked in cotton (Elvira et al., 2010). The adult insects were kept in cages for 20 days, with daily feed replacement. After this period, they were removed from the cages, as well as the filter paper, for analyzing the presence of eggs.

## Results

### *B. bassiana* Conidia

#### Biocompatibility Assays Between the Biopolymers and *B. bassiana*

The assay allowed the calculation of the Biological Index (Gonçalves Diniz et al., 2020), assigning different levels to fungal development stages, quantified by the impacts of encapsulating agents to the microbial growth medium. Table 3 illustrates the results in the biocompatibility evaluation among the encapsulating agents and *B. bassiana*.

**Table 3:** Results of the biocompatibility evaluation between *B. bassiana* conidia and encapsulating agents in PDA growth medium.

**Biopolymer (% w/v)**	**Microbial growth (cm)**	**Sporulation (×10^**7**^)**	**Germination**	**BI^**a**^**	**Classification**
Control	1.50 ± 0.18	4.12 ± 1.10	100 ± 0.00	-*o*-	-*o*-
Corn starch 1%	0.90 ± 0.22	3.77 ± 0.67	105 ± 2.50	78.0	Compatible
Corn starch 2%	1.10 ± 0.13	3.00 ± 1.20	73.0 ± 1.20	73.1	Compatible
Soy oil 1%	1.00 ± 0.11	3.63 ± 0.92	99.0 ± 2.30	79.1	Compatible
Soy oil 2%	0.90 ± 0.08	4.03 ± 1.40	91.0 ± 1.80	79.4	Compatible
Lignin 1%	0.10 ± 0.03	2.72 ± 0.66	77.0 ± 1.90	39.2	Toxic
Lignin 2%	0.00 ± 0.03	0.77 ± 0.45	3.00 ± 0.30	8.33	Toxic
Humic acid 1%	1.00 ± 0.23	3.37 ± 0.56	8.00 ± 0.50	67.3	Compatible
Humic acid 2%	1.20 ± 0.19	0.15 ± 0.06	0.00 ± 0.00	38.7	Toxic

a *Biological Index; The BI values for product classification were arranged as follows: (a) BI ≤ 41: Toxic (T); (b) BI ≥ 42 and ≤66: Moderately toxic (MD); and (c) BI > 66: Compatible (C)*.

Microbial development after 72 h is illustrated in Supplementary Figure 2. The results indicated that *B. bassiana* was resistant to the encapsulating agents in almost all evaluated concentrations, suffering little or no influence during its life cycle. Exceptions in its biocompatibility were observed for lignin (in both dosages, 1% and 2% w/v) and humic acids at a concentration of 2% (w/v).

### Microencapsulation of *B. bassiana*

#### Spray Drying: Optimization Process

In this experiment, we observed that, independent of the temperatures we assessed, the germination and sporulation of *B. bassiana* were maintained. We also noted that we obtained the best conidia recovery (w/w) at the highest evaluated temperature (120°C). The data suggested an accumulation of conidia per gram of powder due to moisture removal from the conidia in the non-formulated commercial product. The conidium quantification data is also displayed in Supplementary Figure 3. This experiment showed that aqueous fungal dispersions withstood temperature exposure in the spray dryer.

Subsequently, we performed the Spray dryer parameter optimization through a full factorial design 2^4^ (Table 2). This optimization was performed with an aqueous fungal solution without encapsulating agents to find the best operational parameters for the recovery of conidia per gram.

**Figure 1:**
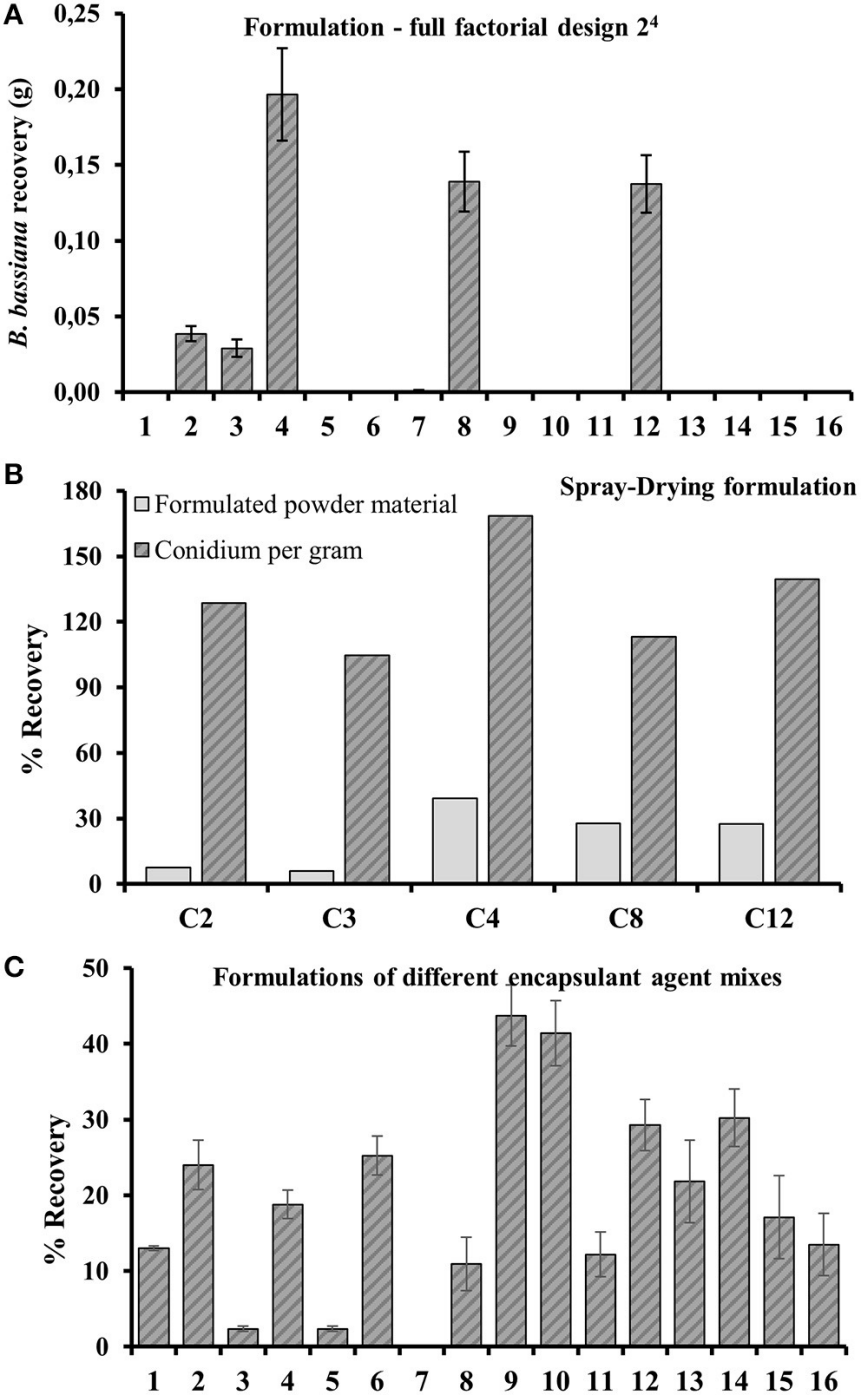
**(A)***B. bassiana* powder recovery (grams) to the 16 spray drying experiments within the full factorial design 2^4^ in Table 2. **(B)** Recovery of total conidia in powder and conidia per gram of powder after spray drying conditions (Conditions 2, 3, 4, 8, and 12 from Table 2). **(C)** Powder recoveries for spray drying formulations in Table 1 after their optimized configuration. Spray drying parameters concerning experiment 4 of full factorial design 2^4^.

Conditions 4, 8, and 12 were more efficient for drying *B. bassiana* in terms of powder recovery (Figure 1A).

**Figure 2:**
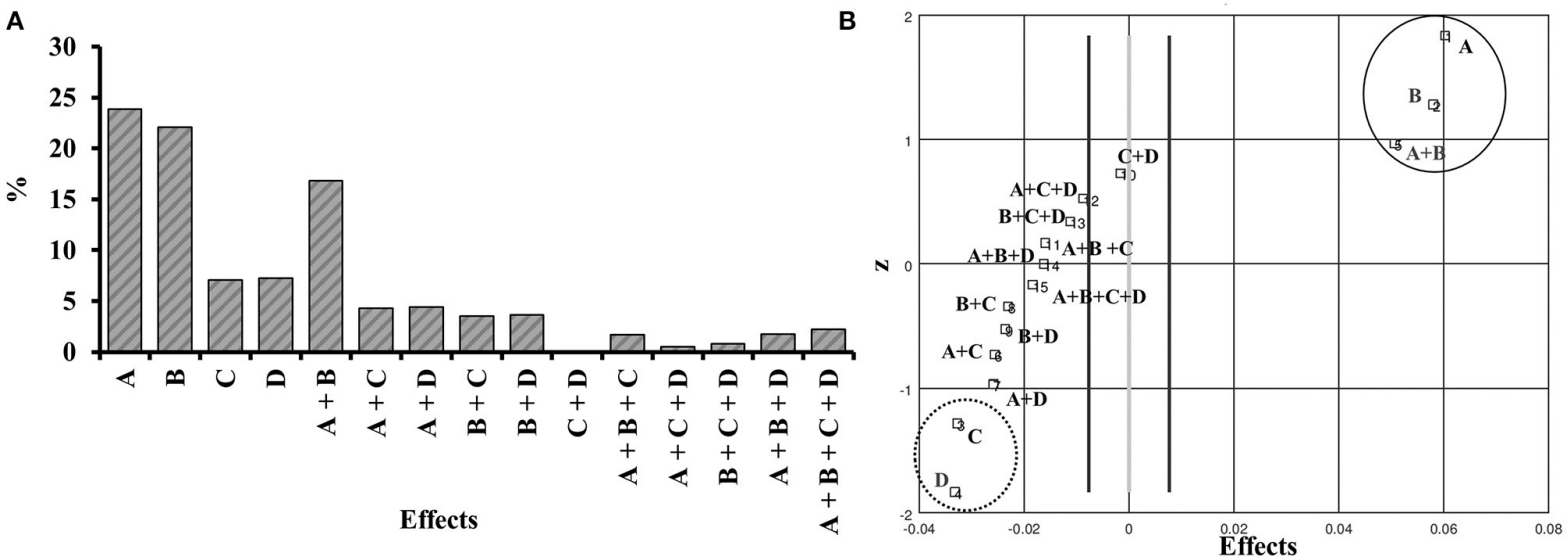
Percentage of primary effects and interactions **(A)**, and distribution of effects **(B)**. A: Inlet temperature; B: Feed flow rate; C: Aspirator rate; D: Air injection flow.

We calculated the effects of each variable from the powder recovery data, considering primary and interaction effects. The experimental variance, experimental error, and effect errors were calculated by effect in a percentages analysis using a confidence level of 95%, with 16 degrees of freedom and a *T*-value = 2.1. These statistical parameters were calculated according to Pereira and Pereira-Filho (2018) and summarized in Supplementary Table 1. The effects percentage and probability can be analyzed in Figure 2.

Effects in percentage analysis indicated that the variables of greatest importance were inlet temperature (24%), feed flow rate (22%), as well as their secondary interaction (inlet temperature/feed flow rate, 17%). The representativeness of both variables was 63% in the total of observed effects. The effect of aspirator rate and air injection flow variables corresponded to only 14% of the total effects. Inlet temperature and feed flow rate variables, plus their interaction demonstrate a positive effect on recovery by spray drying. On the other hand, the aspirator rate and air injection flow variables showed a negative effect. The response surface graphs indicate that the best probability of obtaining good yields in experimental conditions would be an inlet temperature and feed flow rate at maximum levels (+1). In contrast, the aspirator rate and air injection flow should be the opposite, at low levels (−1) (Supplementary Figures 5–7).

Experiment 12 (Table 2) was carried out at the high level of inlet temperature and feed flow rate and low level of the aspirator rate, presenting satisfactory yield and quantity of conidia per gram of powder product. The obtained product in this experiment, however, showed difficulties in handling, with high container wall adhesion and a high humidity rate, probably because it was prepared using a high level of air injection flow. Therefore, we defined the parameters for experiment 4 (Table 2) as optimal for *B. bassiana* conidia spray-drying microencapsulation when using encapsulant agents. The inlet temperature application at the established high level (120°C, +1) has a relationship between reducing inject air moisture and increasing evaporation power.

The inoculation of spray-dried samples in the PDA growth medium showed a greater conidia concentration per gram of dried powder than the starting commercial product. This increase may be associated with the withdrawal of the wet volume from the medium and conidial dehydration. In fact, dehydration of the conidia prolongs their survival by inhibiting their germination and reducing metabolism to a minimum, thus decreasing loss during the drying process and improving the stability of the fungus during storage (Horaczek and Viernstein, 2004). Therefore, experiment 4 (Table 2) was the best condition regarding recovery and the number of conidia per gram of dried product (Figure 1B).

The operational parameters of experiment 4 assigned to the spray dryer were applied in the encapsulation agent evaluation (Table 1). The average recovery of formulations loaded with *B. bassiana* and encapsulating agents after spray drying was 19.7 ± 13.48% (17.04–22.36%) in the 16 formulations described in Table 2 (Figure 1C). The best spray drying recoveries were observed for formulations using a mix of encapsulating agents. Under these conditions, we obtained recoveries of 44, 41, 30, 29, 24, and 21% (w/w) to formulations 9, 10, 14, 12, 2, and 13, respectively (Figure 1C). Individually, among the best recoveries, 70% of them contained lignin, 40% cellulose, 50% soy oil, 70% humic acids, 40% corn starch, and 60% sodium alginate.

#### Preparation and Analysis of Capsules Loaded With *B. bassiana* by Ionic Gelation

After preparing the particles loaded with *B. bassiana* by ionic gelation, we obtained powder material recoveries close to 100% for all formulations (Table 1). All formulations and controls were inoculated in PDA growth medium in order to quantify the number of conidia per gram of formulated powder product. Formulations 3, 8, 12, and 16 of the factorial design (Table 1) for ionic gelation showed reduced viability regarding the non-formulated control; however, superior to most of the ones obtained through spray drying.

#### Considerations About Spray Drying and Ionic Gelation

The products obtained by both processes were analyzed for growth and fungal release for up to 96 h. We observe some similarities and a few differences between the capsules obtained by spray dryer and those obtained by ionic gelation. In the first 48 h, we observed that the formulations 1, 5, and 13 showed the release of hyphae for both preparation processes. However, spray-dried capsules 3 and 11 showed a growth onset, which was not observed for the same capsules obtained by ionic gelation. On the other hand, gelation formulations 6, 9, and 10 had hyphae growth, which did not occur for the same spray-dried formulations. Formulations 2, 4, 7, 8, 12, 14, 15, and 16 did not present fungal release in the observed period of 48 h.

After 72 h, some formulations showed intense sporulation. We highlight that the importance of spore release in the final stage of the insect's infectious process when analyzing virulence. The formulations with intense sporulation for both techniques were 1, 5, and 13. Among the formulations that had not shown a release start within 48 h, 2, 4, 7, 8, 12, 14, 15, and 16 had no posterior hyphae growth. The only exception was formulation 7, which showed the beginning of development, but only those from the ionic gelation process. Formulations 3 and 11 showed intense sporulation when obtained by spray dryer, but hyphae only developed in those prepared by ionic gelation. A reverse result was observed for formulation 6 and 10, which showed sporulation when obtained by ionic gelation and the beginning of hyphae growth by spray-drying. Formulation 9 also showed sporulation only when obtained by ionic gelation without any fungal development by spray drying.

**Figure 3:**
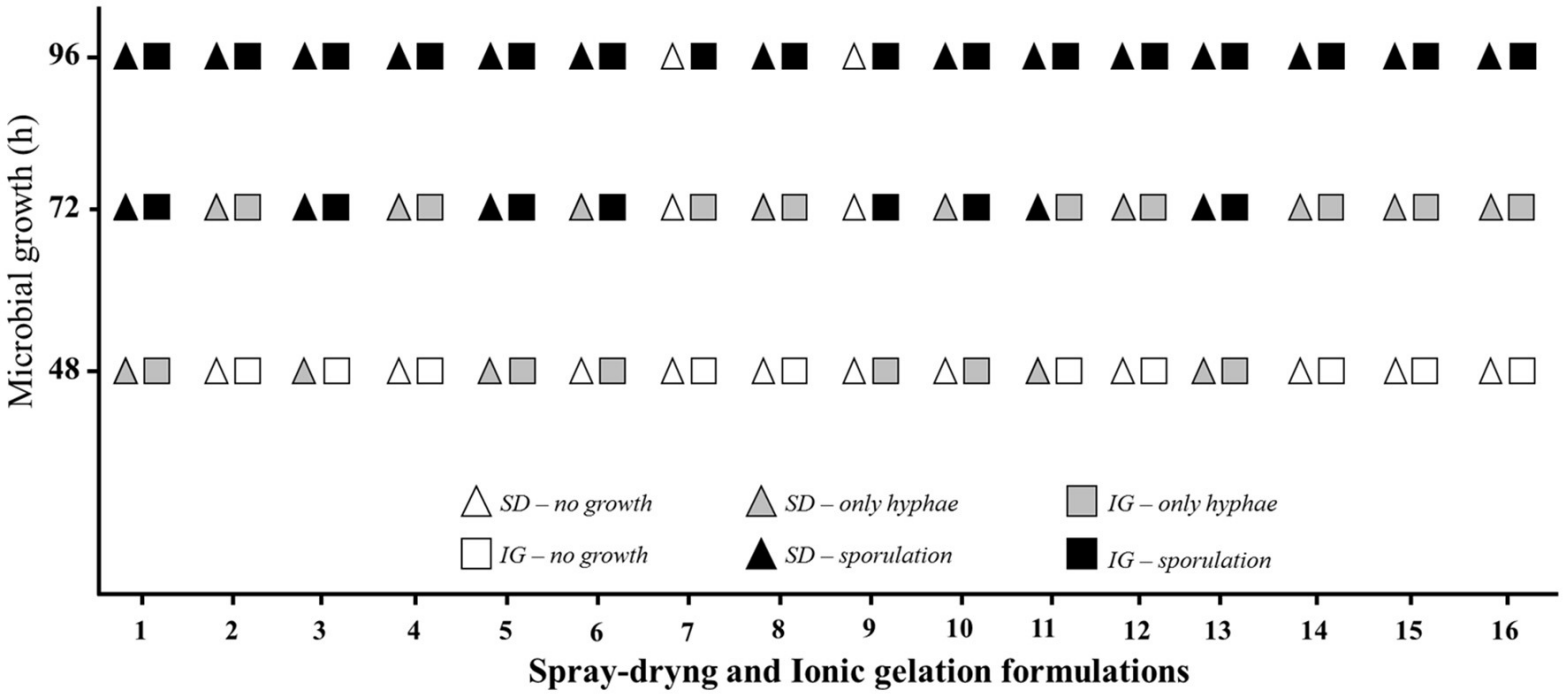
Cultivation of microencapsulated *B. bassiana* by spray dryer and ionic gelation in Potato-Dextrose-Agar growth medium for 96 h. SD, spray-drying; IG, ionic gelation.

After 96 h, all formulations showed intense sporulation, except for formulations 7 and 9 obtained by spray drying, which did not start the fungal release. These formulations were kept for an additional period. After 10 days, there was still no observed fungal development. Figure 3 summarizes what was observed during the cultivation period of 96 h.

We observed that 37.5% of the formulations prepared by ionic gelation and 31.25% of those by spray drying started rapid release, progressing to intense sporulation within 72 h. In comparison, 62.5% of ionic gelation formulations and 56.25% of spray dryer formulations only started the release process after 72 h. At the end of the observed period, we observed that 100% of the formulations obtained by ionic gelation showed intense sporulation, against 87.5% of those by spray drying. A total of 12.5% of the formulations prepared by spray drying did not show any form of growth or fungal release.

### Aqueous Dispersion, Thermal Stability, and UV-Light Protection of Formulated *B. bassiana*

The spray drying and ionic gelation formulations that showed the best results during the viability assays were subjected to thermal and UV-light analyses. From spray drying, we select formulations 1, 3, 5, 6, 8, 9, 11, 13, and 15; from ionic gelation, we chose all 16 formulations. As a variable answer, we once again monitored microbial viability. The results are described in Table 4. Images of microbial development after the thermal stress and UV-light assays are illustrated in Supplementary Figure 8. The thermal stability assays were carried out at 60°C, and UV-light protection was evaluated using an exposition period of 48 h for both spray drying and ionic gelation. Unprocessed conidia (commercial) presented limited development in the growth medium after thermal and UV-light exposition. Conidia processed by spray drying without the use of encapsulating agents (Formulation 1, Table 1) showed reasonable rates of vegetative and germinative growth, with an intense presence of spores (Table 4). Similarly, Formulation 1 obtained by ionic gelation (Table 1), also without encapsulating agents, presented thermal resistance, but not to UV-light.

**Table 4:** Microbial growth analysis of microencapsulated *B. bassiana* exposed to UV-light and controlled temperature (Formulation codes are listed in Table 1).

**Spray drying formulations**																					
	**Formulation code**	**1**		**3**		**5**		**6**		**8**		**9**		**11**		**13**		**15**		**CF**	
	**Fungal development**	**YH**	**HG**	**YH**	**HG**	**YH**	**HG**	**YH**	**HG**	**YH**	**HG**	**YH**	**HG**	**YH**	**HG**	**YH**	**HG**	**YH**	**HG**	**YH**	**HG**
Conditions	Control UV I	++	++	++	++	++	++	++	++	++	++	++	+	++	++	+	+	++	++	+	+
	Control UV II	++	++	++	++	++	++	++	++	++	++	++	+	++	++	+	+	+	+	+	–
	UV (48 h)	++	++	++	++	++	+	++	+	–	–	++	+	++	++	+	+	++	–	+	–
	60°C (4 h)	++	++	+	+	++	+	++	++	++	++	+	–	+	–	–	–	++	+	+	-
**Ionic gelation formulations**																					
	**Formulation code**	**1**		**2**		**3**		**4**		**5**		**6**		**7**		**8**		**CF**			
	**Fungal development**	**YH**	**HG**	**YH**	**HG**	**YH**	**HG**	**YH**	**HG**	**YH**	**HG**	**YH**	**HG**	**YH**	**HG**	**YH**	**HG**	**YH**	**HG**		
Conditions	Control UV I	++	++	++	++	++	++	++	++	++	++	++	++	++	++	++	++	+	+		
	Control UV II	++	++	++	++	++	++	++	++	++	+	++	++	++	++	++	++	+	+		
	UV (48 h)	++	++	–	–	++	++	++	++	++	–	++	++	++	++	++	++	+	-		
	60°C (4 h)	–	–	++	++	–	–	++	++	–	–	++	++	–	–	++	++	+	-		
	**Formulation code**	**9**		**10**		**11**		**12**		**13**		**14**		**15**		**16**					
	**Fungal development**	**YH**	**HG**	**YH**	**HG**	**YH**	**HG**	**YH**	**HG**	**YH**	**HG**	**YH**	**HG**	**YH**	**HG**	**YH**	**HG**				
Conditions	Control UV I	++	++	++	++	+	+	++	+	++	++	++	++	++	++	++	++				
	Control UV II	++	+	++	++	+	+	++	+	++	++	++	++	++	++	++	++				
	UV (48 h)	++	–	++	+	–	–	++	+	–	–	++	++	++	++	++	++				
	60°C (4 h)	++	++	++	++	–	–	+	–	–	–	++	–	++	+	++	+				

*YH, young microbial hyphae; HG, hyphae showing germinative structure; CF, Commercial formulation, unprocessed commercial conidia under no UV or temperature treatment; Control UV I = conidia without exposure to UV radiation kept at 20°C for 48 h; Control UV II = conidia without exposure to UV radiation, however, they were kept inside the chamber during all 48 h of the assay; “++”: vegetative and germinative growth in BDA culture medium after abiotic stress, covering an area larger than 50% of the Petri dish; “+”: vegetative and germinative growth in BDA culture medium after abiotic stress, covering an area <50% of the Petri dish; “–”: no observed vegetative and germinative growth in BDA culture medium after abiotic stress*.

In general, all previously selected formulations prepared by spray drying and ionic gelation had gains in thermal stability and UV-light protection compared to unprocessed material (Table 4). Therefore, we chose formulated products with good results on yield and stability for solubility and biological assays such as formulations 1, 3, 5, and 11, obtained by spray dryer and 4, 6, 8, 10, 15, and 16, by ionic gelation. Formulation 8 prepared by spray dryer using lignin was also selected for the biological assays.

During the stability evaluation, the encapsulating agents that stood out were lignin for ionic gelation assays, and cornstarch in both processes. Starch was present in three (1, 3, and 5) of the four selected formulations prepared by spray drying, while lignin was present in five (4, 6, 8, 10, and 16) of the six chosen formulations obtained by ionic gelation. Formulation 15, obtained by ionic gelation without lignin, showed lower germination growth than the others prepared by the same process.

We noted that the formulations obtained by ionic gelation showed a higher level of UV-light resistance, as 56% presented high sporulation against 33% of those obtained by spray drying. However, both showed similar growth rates concerning temperature, with 33% of spray drying formulations growing after thermal exposure against 37.5% of ionic gelation formulations. Among the nine ionic gelation formulations with the highest sporulation index (1, 3, 4, 6, 7, 8, 10, 14, and 16) obtained after UV-light exposure, only three (1, 3, and 7) had no presence of lignin. On the other hand, the only formulation with lignin obtained by spray drying (8) did not show resistance to UV-light. Such a finding may indicate that the ionic gelation encapsulation process maintains the lignin's UV-light protection ability. Other intrinsic factors to powder formulation are related to the resistance presented by spray drying formulations, not necessarily due to the presence of lignin.

Formulations 1, 3, and 8 obtained by spray drying showed a fast dispersion rate, compatible for use in the field when added in an aqueous phase at a concentration of 1.0 mg·ml^−1^. Formulations 5 and 11 formed small clusters in an aqueous medium which persisted even after hard stirring. The commercial conidia in powder not processed by spray drying presented problems in dispersion stability, producing precipitates soon after the stirring in an aqueous medium.

In general, we observed a loss of thermal stability and UV-light protection in dry powder formulations when analyzing *B. bassiana* conidia after dispersion in an aqueous medium. The spray drying formulations 1, 8, and 11 in suspension maintained the germinative and vegetative growth after the 48 h UV-light protection assay; however, they lost viability after 4 h of exposure at 60°C. Formulations 3 and 5 did not present germination after UV-light protection and temperature assays in aqueous suspension. The unformulated commercial product did not show germination even after simple dispersion in an aqueous medium and maintenance at room temperature for 4 h. These results highlight the stability gain of *B. bassiana* when formulated by spray drying.

No product obtained by ionic gelation showed dispersion capacity in aqueous media. The ionic gelation process generated larger particles than spray drying, presenting limitations for dispersion in an aqueous medium. The spray-dried particles showed an average size on a scale of micrometers while those by ionic gelation were on a scale of millimeters (Supplementary Figure 9).

### Biological Activity of Formulated *B. bassiana* Against *S. cosmioides*

After 23 days of exposing *S. cosmioides* to a diet containing the *B. bassiana* formulated products, we observed an average mortality of 66% (±0.05%) and 76% (±0.07%) for the products prepared by spray drying and ionic gelation, respectively. The average values were similar to the positive control. In this work, the *S. cosmioides* surviving pupae were kept in an incubator chamber for analysis at the adult stage and grouped into three different sets: (1) insects exposed to formulations prepared by spray drying, (2) insects exposed to formulations prepared by ionic gelation, and (3) a control group unexposed to microorganisms. At this stage, we observed that after 13 days, the control group showed an adult outbreak rate of 94.1% for the formed pupae. Although the average total mortality rate of caterpillars to formulations prepared by ionic gelation (76%) was higher than that observed for spray drying (66%), the former presented a smaller number of viable adults at the end of the experiment. These results confirm the maintenance of *B. bassiana* virulence after spray drying formulation (even when using high temperatures as high as 120°C) and ionic gelation.

We also observed that adults formed in the treatments with spray-dried products did not show oviposition during their reproductive cycle. Several caterpillars treated with spray-dried products did not finish their development process. On the other hand, the treatments by ionic gelation products showed a smaller number of adults than spray drying, but they presented oviposition. However, these eggs did not hatch. This result indicates that the low number of viable adults remaining was sufficient for oviposition but not for eggs fertilization and life cycle maintenance. This observation reinforces the hypothesis that due to the small particle size of conidia obtained by spray drying, the insects could ingest them, affecting its development. At the end of the life cycle, the spray drying and ionic gelation products performed similar results to control on *S. cosmioides*, as indicated in Table 5. The Supplementary Figures 10–12 illustrate some *S. cosmioides* during their life cycle after exposition to our formulations.

**Table 5:** Impacts of spray drying microencapsulated *B. bassiana* by (SD) and ionic gelation (IG) in *S. cosmioides* life cycle stages.

**Formulation**	**Caterpillar mortality (%)**	**Formed pupae (%)**	**Average pupal weight ±σ (g)**	**Total mortality (%)**
SD 1	30	30	0.320 ± 0.015	70
SD 3	40	40	0.370 ± 0.023	60
SD 5	30	30	0.290 ± 0.024	70
SD 8	20	40	0.300 ± 0.002	60
SD 11	50	30	0.360 ± 0.034	70
IG 1	70	30	0.390 ± 0.025	70
IG 4	70	20	0.310 ± 0.000	80
IG 6	50	20	0.290 ± 0.007	80
IG 8	40	30	0.340 ± 0.014	70
IG 10	0	10	0.370 ± 0.000	90
IG 15	50	30	0.280 ± 0.042	70
IG 16	40	30	0.320 ± 0.000	70
*Control C–*	0	100	0.360 ± 0.037	0
*Control CA–*	0	100	0.340 ± 0.039	0
*Control C+*	50	0	*-o-*	100

*SD, caterpillars subjected to assay with products formulated by spray drying; IG, caterpillars subjected to assays with products formulated by ionic gelation; C+: positive control, containing unformulated B. bassiana; C–, negative control, treatment free of entomopathogenic microorganisms; CA–, negative control, entomopathogenic treatment free of microorganisms, however, containing alginate. Numbers are the formulation as described in Table 1; σ, standard deviation*.

## Discussion

The use of biocontrol agents in the field shows several advantages, such as reducing toxic compounds in the environment and a better balance to the plant-insect microsystem. The popularization of biological control agents, however, depends on the optimization of processes and/or formulations that reduce their costs and increase their stability, ensuring easy application and maintenance in the field. Thereby, the stability of the biocontrol agents should be one of the pillars in their development, since it has impacts on logistic costs, storage protocols, and field application methods.

Microencapsulation is a process that can increase the stability of biological agents, providing a protection and releasing them from the matrix in a controlled manner in the environment. Several polymers are described as viable matrices for encapsulating different chemical classes of compounds (Vijeth et al., 2019). It is crucial for microorganisms that the encapsulating polymer be biocompatible, not reducing its viability and/or virulence, and acting as a protective and inert carrier, for its eventual controlled release. Therefore, we highlighted three points that should be considered in developing microbial formulations for insect control: (1) stability, (2) biocompatibility to the microorganism, and (3) sustainability of the encapsulating polymer.

As possible encapsulating material, we selected sodium alginate, humic acids, cellulose, corn starch, lignin, and soy oil, all of which were subjected to biocompatibility assays. These materials were chosen due to their low costs, their abundance in the Brazilian territory with some of them actually being waste from other agricultural activities, and all of them being biodegradable and non-toxic products. Cellulose and lignin show high rigidity, tensile strength, and thermal stability (Wandrey et al., 2010). According to Leland and Behle (2005) and Sipponen et al. (2019), lignin can protect against UV-light. Corn starch is easily found with high availability, low cost, and water retention ability (Forssell et al., 2004). Soy oil shows tolerance to thermal stress, has a low cost, and can stick fast to the surface of materials (Leland and Behle, 2005; Wandrey et al., 2010). In turn, humic acids promote resistance and thermal and UV-light stability and are beneficial to soil and plants (Tomaszewski et al., 2011; De Melo et al., 2016).

The exceptions in its biocompatibility that were observed for lignin (in both dosages, 1 and 2% w/v) and humic acids at a concentration of 2% (w/v) could be related to their toxicity. The toxicity of lignin and humic acid is probably related to variation in the pH value due to the change in the composition of the growth medium. The initial pH value of the PDA growth medium was 5.8. However, after adding lignin into the culture medium, the pH rose to 8.2 and 9.3 with 1 and 2% (w/v) of lignin, respectively. Meanwhile, humic acids reduced the pH values to 5.3 and 4.5 with 1% and 2% (w/v) in mass, respectively. Previous studies demonstrate that *B. bassiana* was able to withstand a wide variation in the pH values between 5 and 10 (Padmavathi et al., 2003; Luo et al., 2015). Therefore, the toxicity observed to lignin and humic acid may be related to chemical changes and nutrient availability due to pH variation, resulting in a poor environment and compromising fungal development. With this in mind, we decided to keep lignin among the compounds for the next stages due to its potential properties as an encapsulating agent (biodegradability, photoprotective properties, low cost, etc.) and also its cost efficiency, as it is a waste product from the pulp and paper industry. Moreover, previous studies in our research group showed that lignin, when applied in formulations, was not harmful to *Spodoptera frugiperda* (Costa et al., 2017).

A possible disadvantage to using spray drying to living organisms is the possibility of dehydration with a reduction in biological viability. Therefore, we evaluated the fungal resistance to spray dryer temperatures in the absence of encapsulating agents.

The resistance to thermal degradation during spray drying can be explained by the equipment operating principles. The solvent surface tension protects the compounds present in the formulation during the warm air flow. Heat promotes solvent evaporation after nebulization. An internal cooling occurs during the evaporation process in the micro-drops, since it consumes sensitive heat, turning it into latent heat, keeping the thermo-unstable compounds protected (Santos et al., 2018). Therefore, during the whole process, there is little thermal change inside the droplets, resulting in a low impact on microbial viability (Keshani et al., 2015).

The final product by spray-drying tends to have less moisture, directly impacting the recovery of the powder products (Woo et al., 2007; Santos et al., 2018). The feed flow rate also showed better recovery results when optimized at the highest level. Using a higher feed flow rate, the particle size is influenced due to the presence of a larger volume of dispersion liquid, producing more diluted drops and better-separated particles. The partial system pressure is also affected due to a larger volume of liquid for evaporation in a given period. Moreover, higher feed flow rates reduce the outlet temperature consuming thermal energy during evaporation, which is important for protecting biological samples from temperature exposure (Santos et al., 2018). Therefore, we observed that a higher feed flow rate associated with a higher inlet temperature provided a better recovery index of conidia powder, with a refined appearance and low adherence to the containers.

The formulations that showed the best recoveries were not necessarily those that performed best in the biological assays. We observed that the encapsulating agents that better-influenced powder material recoveries such as lignin and humic acids were also the compounds that showed the worst biocompatibility rates.

The products obtained by spray drying in formulations 1, 3, 5, 11, 13, and 15 presented the best *B. bassiana* growth in the PDA medium. Similarly, the formulations that showed the most significant quantity of conidium per gram of dried products were not prepared using lignin. Only two of them had humic acids in their composition. On the other hand, all formulations with corn starch present as an encapsulating agent showed good results on microbial viability, even when associated with cellulose and alginate, alginate and soy oil, humic acids and cellulose, and humic acids and soy oil. It is relevant to highlight the importance of biological viability analysis together with product development, since formulations with better powder yield results (% w/w) may not necessarily present the best biological activity.

The *B. bassiana* encapsulation by the extrusion of a hydrocolloid alginate solution in a calcium chloride solution is a simple operational process. It consists of mixing the compound/conidia of interest to the alginate solution and dripping it into a gelling solution containing a divalent cation (usually calcium chloride) (Ching et al., 2017). The particle size can be controlled by the extruder nozzle and by the transfer rate. Alginate is a porous trapping matrix allowing the controlled release of the active compound. Moreover, the process does not need high temperatures, collaborating to preserve the microbial viability (Skjåk-Bræk and Draget, 2012; Perullini et al., 2015).

This viability reduction of the formulated products by ionic gelation may be due to a slow-release mechanism of the conidia or loss of viability during the trapping process. Maruyama et al. (2020) encapsulated *Trichoderma harzianum* using alginate. In this work, they observed not only the maintenance of the microorganism virulence but also an efficiency increases in the bioassays. Several studies demonstrate that microbial encapsulation by ionic gelation presents little stress to the entomopathogenic microorganisms (Loomis et al., 1997; Batista et al., 2017; Wenzel Rodrigues et al., 2017).

Thermal stability and UV-light protection are important parameters to be analyzed during the development of biological control products. Thermal and UV-light experiments confirmed an increase in the stability of *B. bassiana*, especially by the spray drying process, which produces dehydrated conidia. The moisture loss as described before, where we observed an increase in the number of conidia compared to the commercial starting product, occurred after the drying process. Mascarin et al. (2016) also demonstrated an increase in the stability and shelf life of *B. bassiana* blastospores after the convection drying process, corroborating our results. Lignin is a polymer with a recognized ability to protect against UV-light (Sadeghifar et al., 2017).

This ability to disperse the conidia formulated by spray drying decreases the need for additives when preparing broth for field application. This gain in aqueous medium dispersion capacity is probably due to the smaller size of the microparticles obtained via spray drying. Peil et al. (2020) demonstrate that the use of lignin in the production of particles loaded with *Trichoderma reesei* spores resulted in a solvent-free system, which could be applied in the field using only water as a vehicle.

The stability of formulated conidia after aqueous dispersion suggests that the spray broth need to be prepared moments before field application. Greenhouse studies are still needed to confirm this. No product obtained by ionic gelation showed dispersion capacity in aqueous media limiting its use as granulated powder.

An *in vitro* biological assay was carried out to analyze the virulence of *B. bassiana* conidia against *S. cosmioides* after formulation. Microbial virulence is associated with genetic factors, such as expression at the level of specific genes, which can change during the microorganisms' exposure to stress (Al Khoury et al., 2019). Therefore, the preparation of formulations in powder using encapsulating agents could change *B. bassiana* virulence due to exposition to stress factors intrinsic to each process.

When exposing *S. cosmioides* to a diet containing the *B. bassiana* formulated products, we observed an average mortality similar to the positive control and other described works in the literature using entomopathogenic fungi in the caterpillar control for the *Spodoptera* genus. Thomazoni et al. (2014) observed a mortality of 44.5% after immersion of *S. frugiperda* into a fungal solution containing different *B. bassiana* isolates. The Ahmed and El-Katatny (2007) work describes the application of *B. bassiana, Trichoderma harzianum*, and *Aspergillus flavus* unencapsulated suspensions at *Spodoptera littoralis* with mortality rates of 90, 80, and 100, respectively at the pupal stage. Bugeme et al. (2008) highlighted that the room temperature variation to non-formulated *B. bassiana* caused a loss in viability and virulence. Moreover, the authors showed that the formulated *B. bassiana* products kept the microbial virulence and were able to control *S. cosmioides*, presenting low viability of caterpillars, pupae, and eggs.

The results observed during the bioassays confirm the maintenance of *B. bassiana* virulence after spray drying formulation (even when using high temperatures as high as 120°C) and ionic gelation. A hypothesis to the differences we observed may be the role of smaller particle diameter obtained in the powder products by spray drying in comparison to ionic gelation. Small particle sizes present favorable features to ingestion and adherence to insects (Stadler et al., 2018; Arthur et al., 2019).

## Conclusions

In this work, we were able to prepare different powder formulations loaded with *B. bassiana* conidia. These materials presented a high mortality rate against *S. cosmioides* in biological assays. Moreover, we also concluded that the formulated materials showed gains in thermal stability and UV-light protection. These results were possible using low-cost materials such as corn starch, soy oil, cellulose, lignin, alginate, and humic acids.

Both spray drying and ionic gelation processes were able to perform the encapsulation of *B. bassiana* conidia. Ultimately, the products obtained by both methods showed similar biological control against *S. cosmioides*; however, the spray-dried powder material showed better aqueous dispersion capacity and smaller storage volume for the same conidium concentration. Nevertheless, the gains in thermal stability and UV-light resistance were similar for both encapsulation techniques. Although alginate microcapsules have better compatibility with encapsulating agents, the final product obtained by spray drying appears to be more advantageous in the context of field application.

## Data Availability Statement

The original contributions presented in the study are included in the article/Supplementary Material, further inquiries can be directed to the corresponding author.

## Author Contributions

MF conceived the study, analyzed and interpreted the data, and critically read and revised the manuscript. AF and RM designed the experiments, acquired, analyzed, interpreted the data, and drafted the manuscript. IR participated in the data discussion. MS and JF participated in the data discussion and with financial support. All of the authors contributed to data analysis, reviewed, and approved the final manuscript.

## Conflict of Interest

The authors declare that the research was conducted in the absence of any commercial or financial relationships that could be construed as a potential conflict of interest.

## Publisher's Note

All claims expressed in this article are solely those of the authors and do not necessarily represent those of their affiliated organizations, or those of the publisher, the editors and the reviewers. Any product that may be evaluated in this article, or claim that may be made by its manufacturer, is not guaranteed or endorsed by the publisher.
