# Supplementary material for: Encapsulation of B. bassiana in Biopolymers: Improving Microbiology of Insect Pest Control
Source: Front Microbiol. 2021 Aug 16;12:704812. doi: 10.3389/fmicb.2021.704812 (PMC8415709; doi:10.3389/fmicb.2021.704812)
Supplement: Supplementary file 1 [file Data_Sheet_1.docx]

Encapsulation of *B. bassiana* in biopolymers: improving microbiology of insect pest control

**Ana Paula Felizatti^1^, Roberta Maria Manzano^1^, Inajá M. Wenzel Rodrigues^1^, Maria Fátima das Graças Fernandes da Silva^1^, João Batista Fernandes^1^, Moacir Rossi Forim^1*^**

^1^Laboratory of Natural Products, Universidade Federal de São Carlos, São Carlos, Brazil.

*** Correspondence:**Prof. Dr. Moacir Rossi Forim
[*mrforim@ufscar.br*](mailto:mrforim@ufscar.br)

Supplementary Material

**Supplementary Figure 1.** Microscopy images of *B. bassiana* and exemplification of the density of fungal structures present, according to the color observed in the growth dish. Esporulation structure and conidia at 100× (**A**) and 400× (**B**) magnification, respectively. Hyphae dish (**C**); Hyphae under a microscope (100×) without sporulation structures (**D**); Growth dish with sporulation points (**E**); Microscopy of low-density sporulation structures (100×) (**F**); Dish with thickening of sporulation points (**G**); Microscopy of dense sporulation structures (100×) (**H**).

**
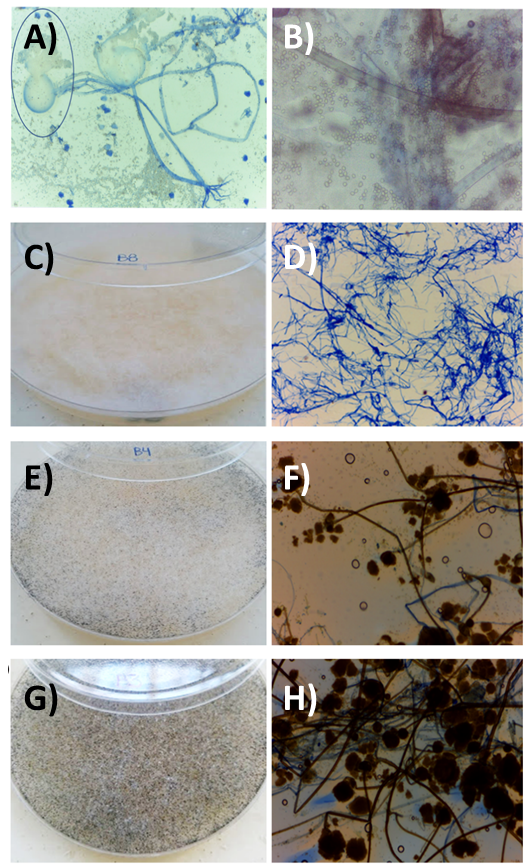
**

**Supplementary Figure 2.** Cultivation dishes from the biocompatibility assay with different concentrations of compounds (1 or 2%, w/v) added at the Potato-Dextrose-Agar (P.D.A.) growth medium. Control: P.D.A. growth medium.


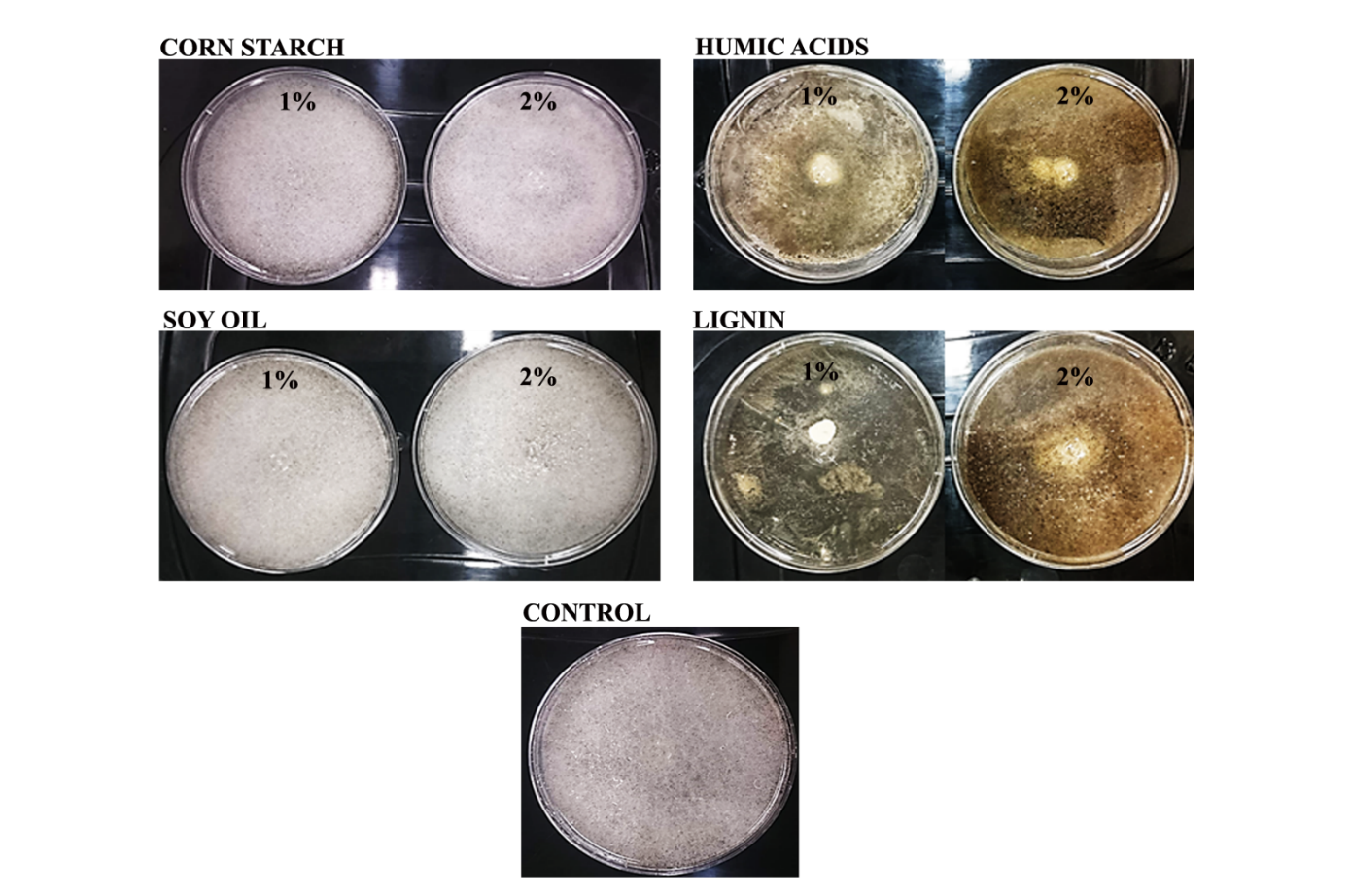


**Supplementary Figure 3.** Conidia recovery per gram of dry powder after exposure to different Spray-dryer inlet temperature conditions.





**Supplementary Figure 4**. Exemplification of powder conidia recovery after exposure to Spray-dryer optimization parameters by full factorial design 2^4^.


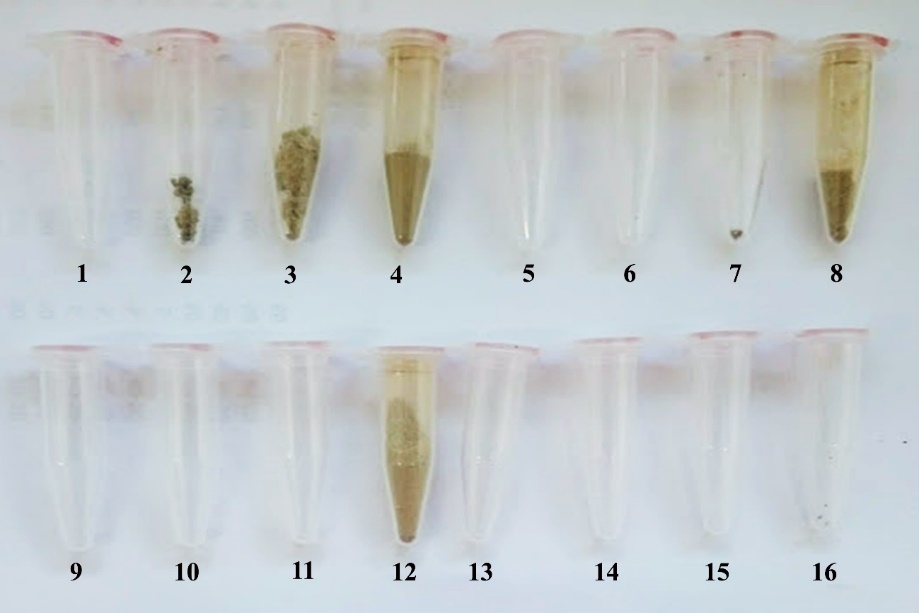


**Supplementary Figure 5**. Surface graph considering the inlet temperature and feed flow rate to estimate the best parameters to powder's recovery in grams.


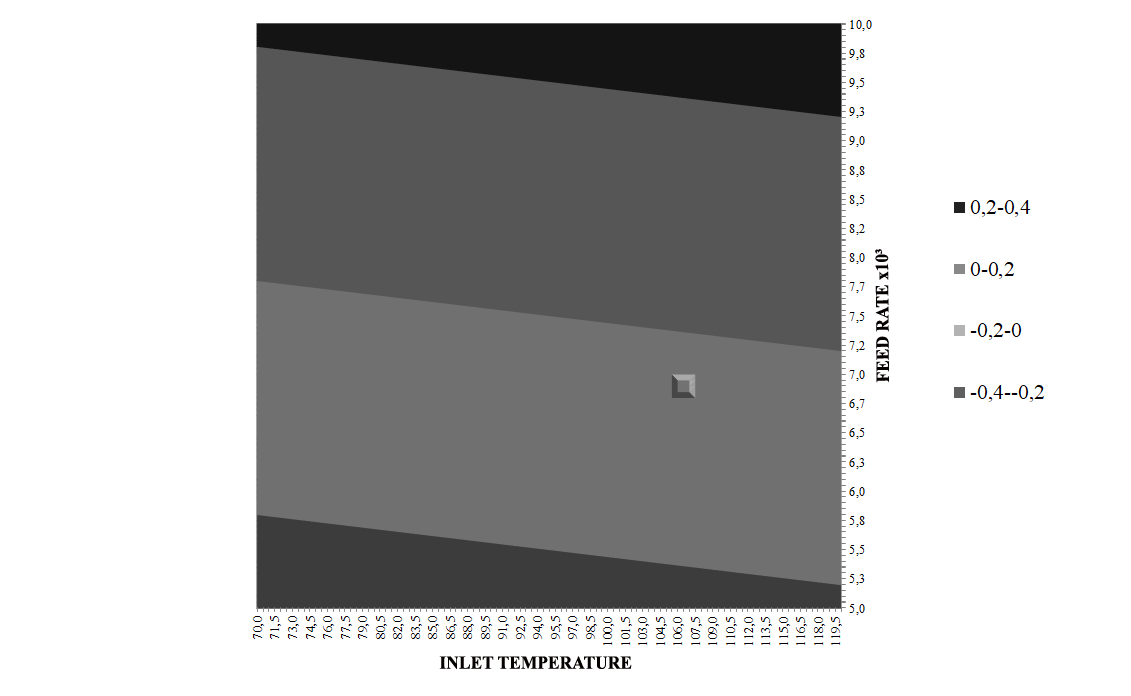


**Supplementary Figure 6.** Surface graph considering the inlet temperature and aspiration rate to estimate the best parameters to powder's recovery in grams


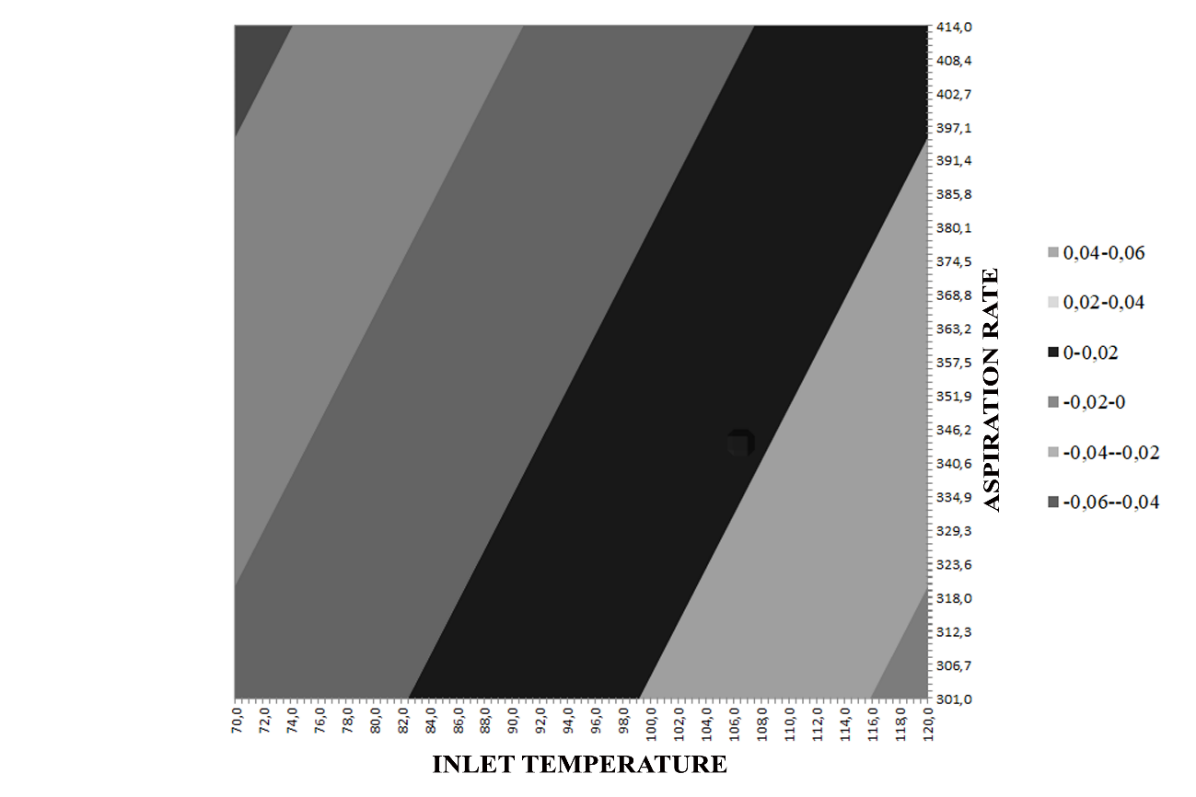


**Supplementary Figure 7.** Surface graph considering the inlet temperature and air injection flow to estimate the best parameters to powder's recovery in grams


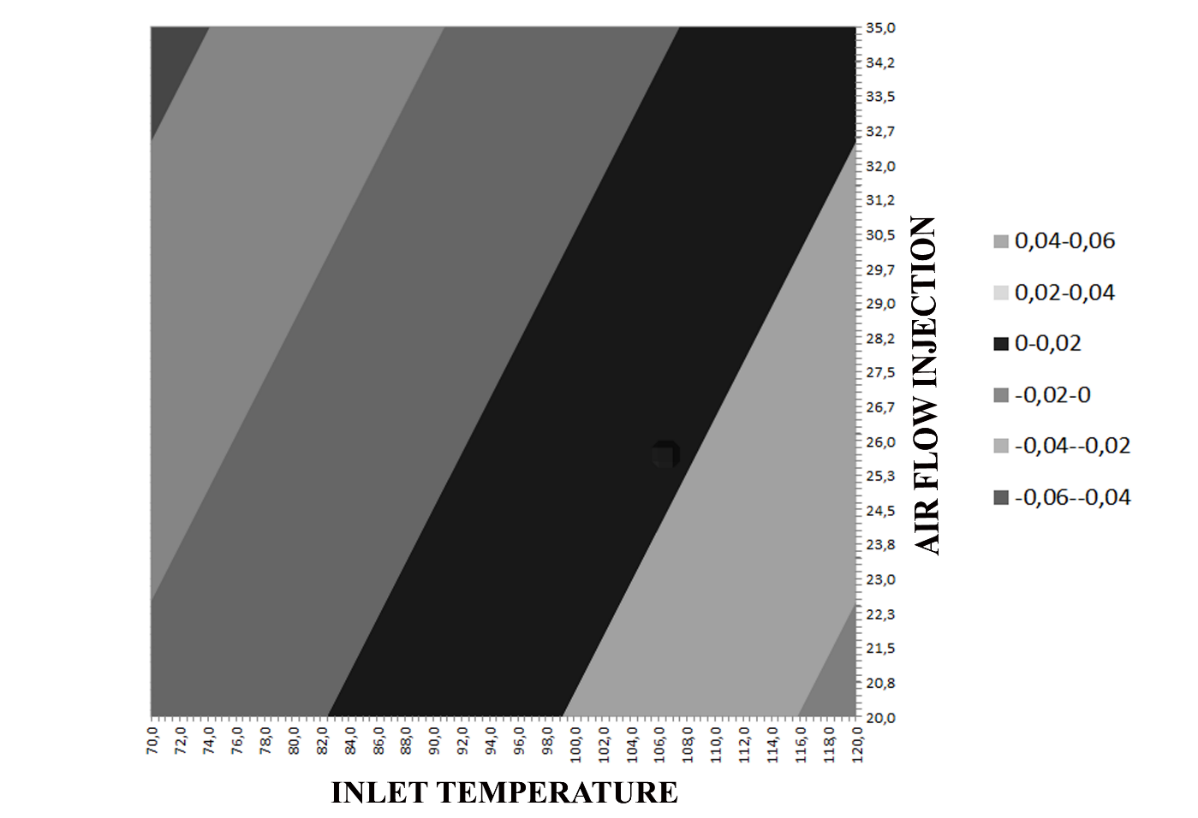


**Supplementary Figure 8**. Cultivation dishes in PDA + pentabiotic after temperature stress and UV-light assays of Spray-drying formulations. **A)** Inoculation after exposure to UVlight (48 h) *Left:* Commercial powder, presence of few hyphae and absence of germination and sporulation. *Right:* Atomized conidium powder, with high hyphae presence and occurrence of sporulation. **B)** Formulation 1. *Left:* 48 h UV-light exposure. *Right:* Post temperature stress at 60ºC for 4 h. **C)** Formulation 3. *Left:* 48 h UV-light exposure. *Right*. Post temperature stress at 60ºC for 4 h. **D**) Formulation 5. *Left:* 48 h UV-light exposure. *Right*. Post temperature stress at 60ºC for 4 h. **E)** Formulation 11 – *Left:* 48 h UV-light exposure. *Right*. Post temperature stress at 60ºC for 4 h**. F)** Formulation 8 - after exposure to 60ºC for 4 h.


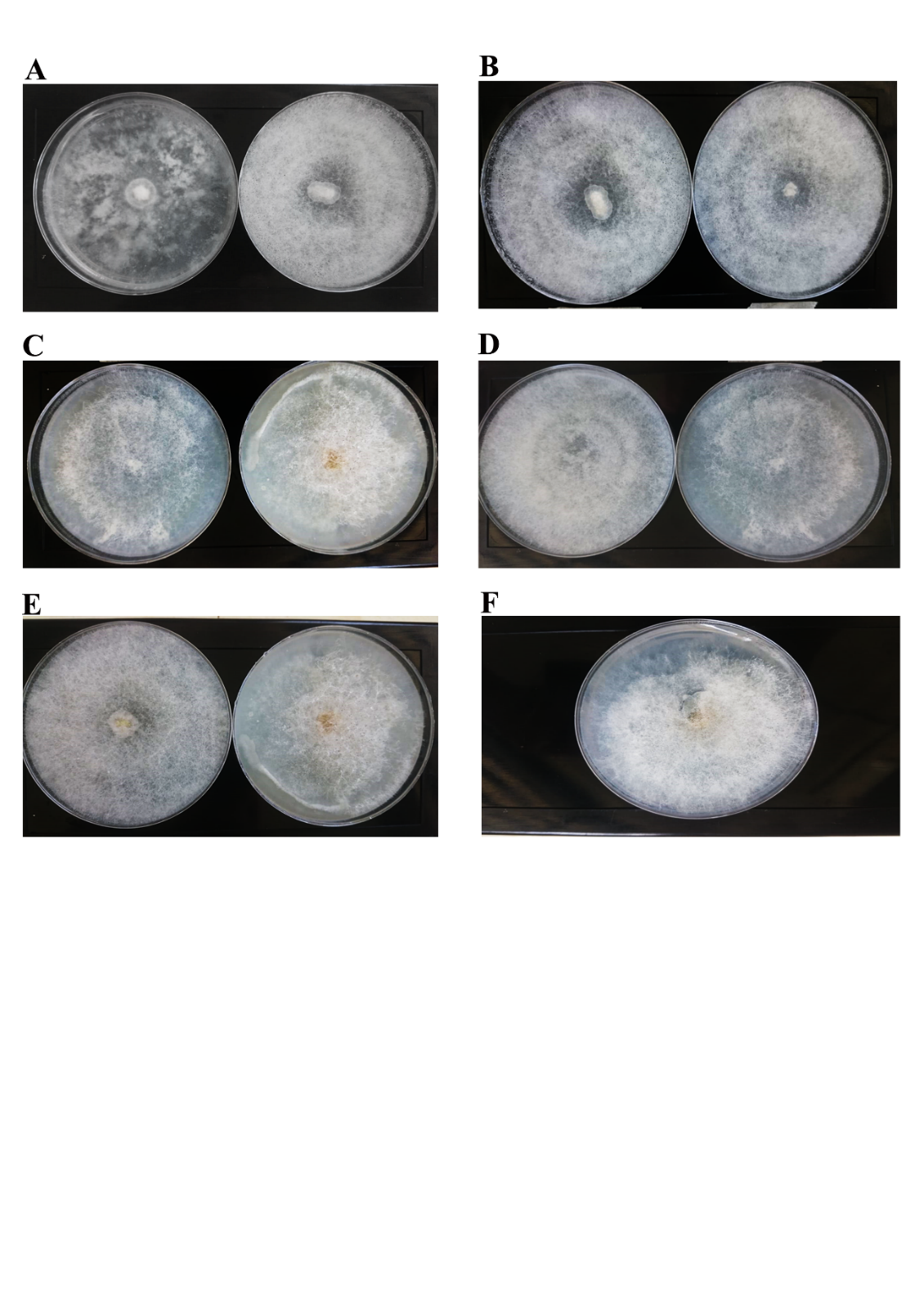


**Supplementary Figure 9.** Example of formulations obtained by different techniques. **A**. In the dishes, obtained by Ionic gelation, in the tubes, by Spray-dryer. **B**. Ionic gelation spheres. **C**. Dehydrated gelling spheres.


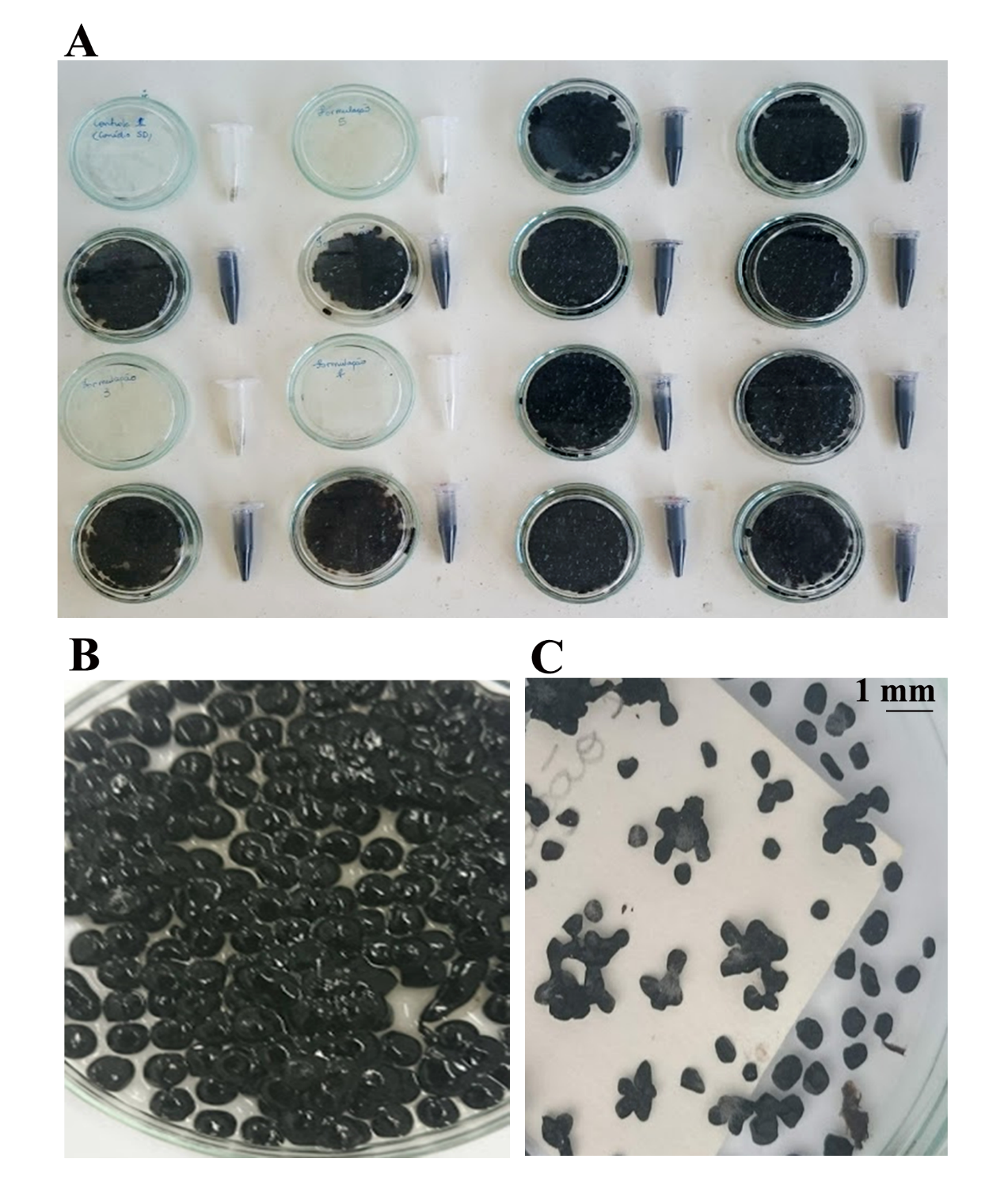


**Supplementary Figure 10.** Specimens of *S. cosmióides* post life cycle exposed to Spray-drying (SD) formulations 1, 3, 5, 8, and 11, indicating harmful changes in the insect cycle, with the low pupal formation


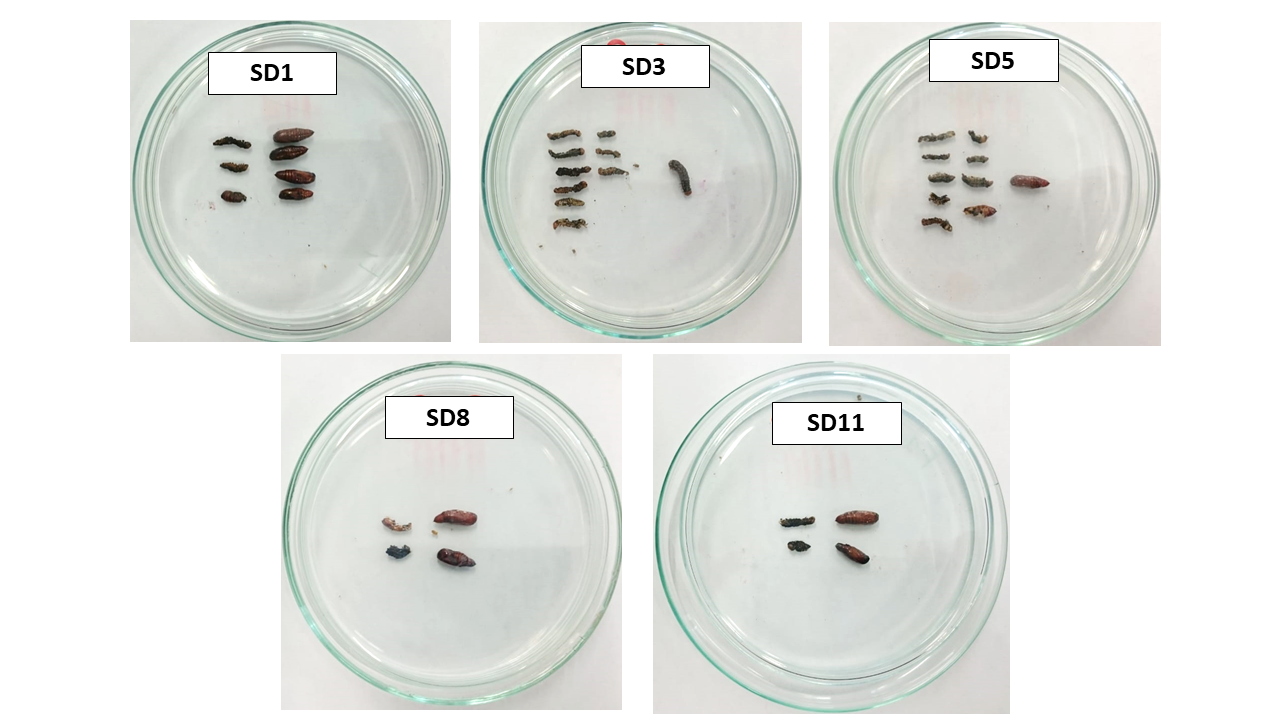


**Supplementary Figure 11**. Specimens of S*. cosmióides* post life cycle exposed to Ionic gelation (SD) formulations 1, 4, 6, 8, 10, 15, indicating harmful changes in the insect cycle, with the low pupal formation


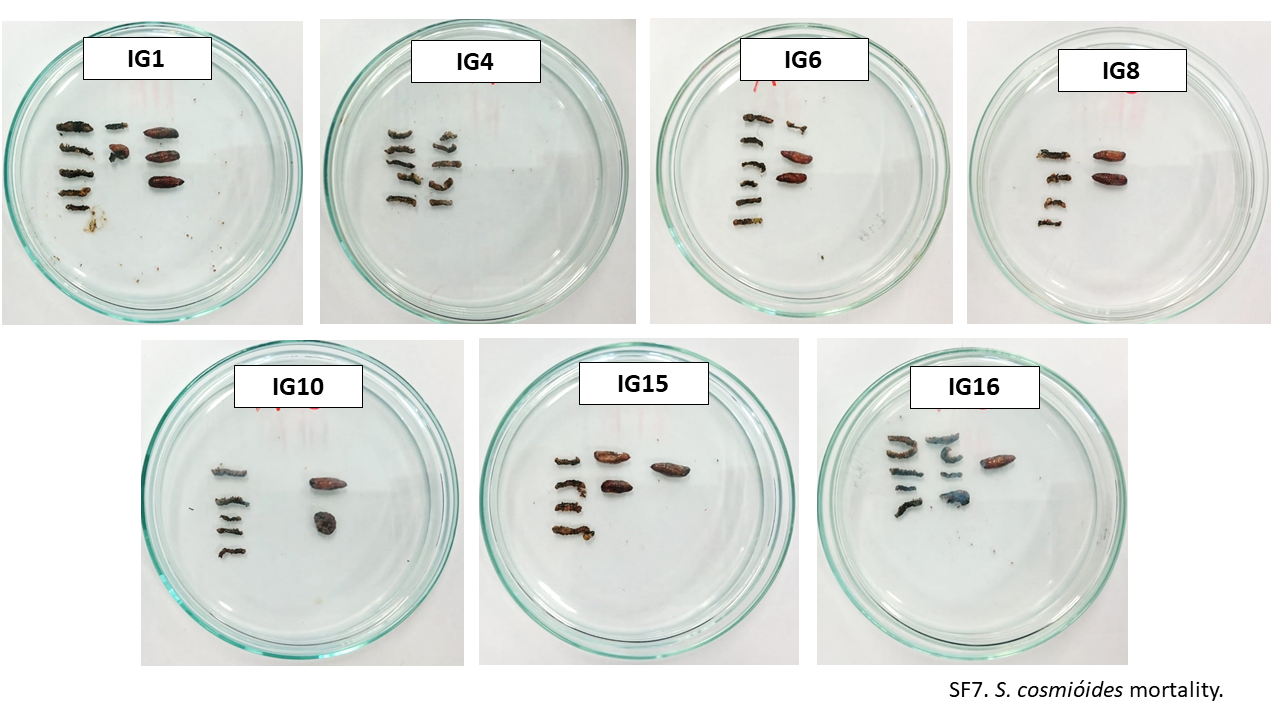


**Supplementary Figure 12.*.*** Specimens of *S. cosmióides* post life cycle under control experiments. C-: No exposure, with complete pupae formation. C(IG) exposure to empty alginate spheres, with majority complete pupae formation. C+ *B. bassiana*: exposure do *B. bassiana* non formulated, with all caterpillars that have not reached the pupal stage


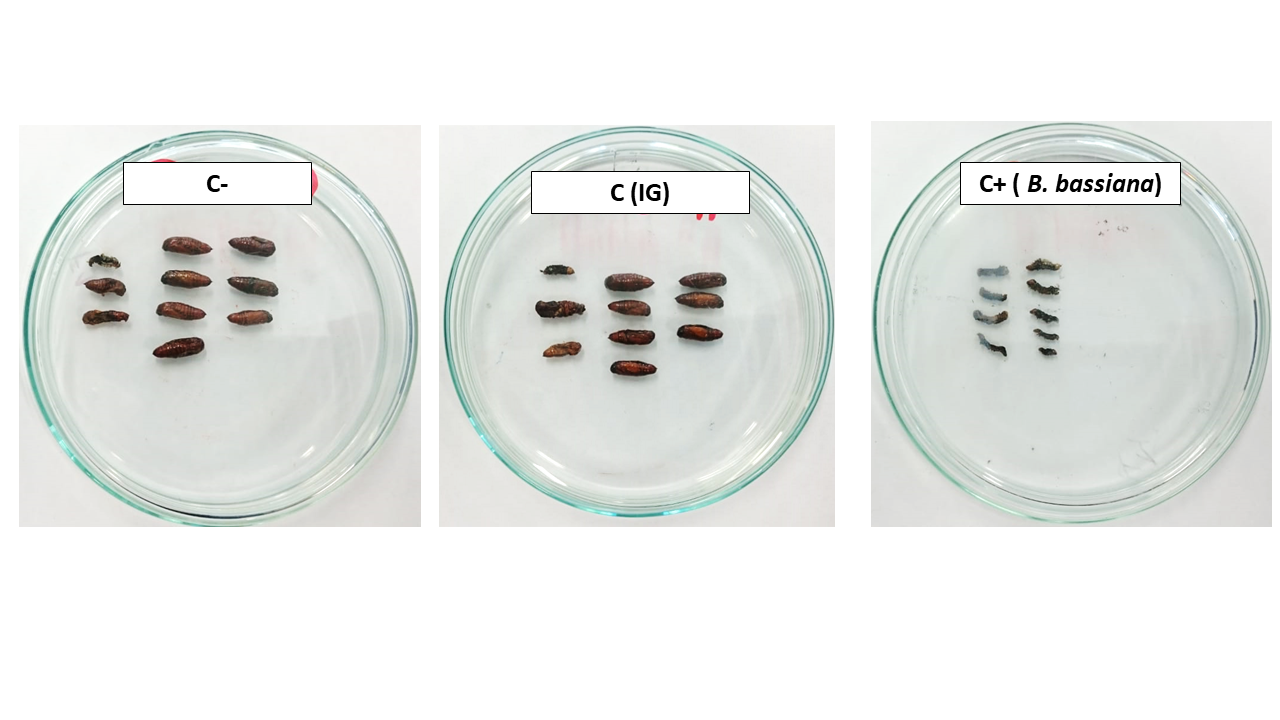


**Supplementary Table** **1.** Statistical parameters of the powder recovery by Spray-dyer optimization concerning the full factorial design 2^4^. R_1_: replicate 1, R_2_: replicate 2, $\bar{x}$ = mean, σ = standard deviation, σ² = variance.

| Spray-Drying  Experiment | *B. bassiana* | | | | |
| --- | --- | --- | --- | --- | --- |
|  | R_1_ | R_2_ | $\bar{x}$ | σ | σ² |
| 1 | 0 | 0 | 0 | 0 | 0 |
| 2 | 0.035 | 0.0420 | 0.0385 | 0.0049 | 2.5 × 10^-5^ |
| 3 | 0.025 | 0.0330 | 0.0290 | 0.0057 | 3.2 × 10^-5^ |
| 4 | 0.175 | 0.2180 | 0.1965 | 0.0304 | 9.2 × 10^-4^ |
| 5 | 0 | 0 | 0 | 0 | 0 |
| 6 | 0 | 0 | 0 | 0 | 0 |
| 7 | 0.001 | 0.0000 | 0.0005 | 0.0007 | 5.0 × 10^-7^ |
| 8 | 0.125 | 0.1530 | 0.1390 | 0.0198 | 3.9 × 10^-4^ |
| 9 | 0 | 0 | 0 | 0 | 0 |
| 10 | 0 | 0 | 0 | 0 | 0 |
| 11 | 0 | 0 | 0 | 0 | 0 |
| 12 | 0.151 | 0.1240 | 0.1375 | 0.0191 | 3.6 × 10^-4^ |
| 13 | 0 | 0 | 0 | 0 | 0 |
| 14 | 0 | 0 | 0 | 0 | 0 |
| 15 | 0 | 0 | 0 | 0 | 0 |
| 16 | 0 | 0 | 0 | 0 | 0 |
|  |  |  |  | *V_exp* | *1.09* × 10^-4^ |
|  |  |  |  | *E_exp* | *1.04* × 10^-2^ |
|  |  |  |  | *E_effects* | *3.68* × 10^-3^ |
